# The efficacy of intermittent pneumatic compression and negative pressure therapy on muscle function, soreness and serum indices of muscle damage: a randomized controlled trial

**DOI:** 10.1186/s13102-021-00373-2

**Published:** 2021-11-13

**Authors:** Szczepan Wiecha, Martyna Jarocka, Paweł Wiśniowski, Maciej Cieśliński, Szymon Price, Bartłomiej Makaruk, Jadwiga Kotowska, Dorota Drabarek, Igor Cieśliński, Tomasz Sacewicz

**Affiliations:** 1 Department of Physical Education and Health in Biala Podlaska, Faculty in Biala Podlaska, Jozef Pilsudski University of Physical Education in Warsaw, Biala Podlaska, Poland; 2 3Rd Department of Internal Medicine and Cardiology, Medical University of Warsaw, 02-091 Warsaw, Poland

**Keywords:** Soreness, DOMS, Recovery, Compression, Vacuum

## Abstract

**Background:**

The study aimed to assess whether intermittent pneumatic compression (IPC) and intermittent negative pressure (INP) would attenuate the muscle damaging effects of eccentric exercise.

**Methods:**

Forty-five healthy males were recruited. Immediately post, 24 and 48 h post eccentric exercise consisting of 100 drop jumps, volunteers randomly received 30-min sessions of intermittent pneumatic compression (IPC, n = 15) or intermittent negative pressure (INP, n = 15), or sham microcurrent (PT, n = 15). Creatine kinase (CK), lactate dehydrogenase (LDH), isokinetic muscle strength, soreness and active flexion of the knee joint were measured after every therapy session.

**Results:**

No significant intergroup differences were observed in biochemical or functional measurements. However, there was an increase in muscle soreness (*P* < 0.05), CK and LDH activity (*P* < 0.05), and a reduction in muscle strength (*P* < 0.05) and range of active knee flexion (*P* < 0.05).

**Conclusions:**

The prescription of IPC and INP did not attenuate the reduction of markers to muscle function or pain perception up to 48 h after muscle damaging exercise. Future research should focus on the potential impact of treatment frequency and duration on muscle recovery.

*Trial registration* The study was retrospectively registered in the Australian New Zealand Clinical Trials Registry (ANZCTR); The trial registration number: ACTRN12621001294842; date of registration: 24/09/2021.

**Supplementary Information:**

The online version contains supplementary material available at 10.1186/s13102-021-00373-2.

## Introduction

Physical activity, especially of high intensity, leads to tissue microtrauma, mainly in the skeletal muscle tissue. This microtrauma is perceived as soreness after exercises and is referred to as delayed onset muscular soreness (DOMS) [1]. Repeated eccentric exercises most commonly induce DOMS, the onset of which usually occurs between 12 and 48 h after heavy exertion [2]. DOMS may be caused by an inflammatory reaction resulting from muscle tissue damage. Damage to the sarcolemma and the muscle cell membrane causes a release of the biochemical markers, such as creatine kinase (CK),which can be used to determine recovery rate [3]. Besides, the release of proinflammatory cytokines leads to an accumulation of leukocytes [2]. Some authors also point to microdamage to peripheral nerves as the reason for the perceived pain and limited functional capacity [4]. Microtrauma leads to prolonged muscular dysfunction and impaired force generation, decreasing athletic performance [5]. Apart from subjective soreness, micro-tears to the muscle tissue also cause proprioception dysfunction and impair subsequent motor skill learning [6, 7]

Fast and optimal muscle recovery after training is significant for athletes, especially during frequent participation in competitive events. Comparative studies show that massage is the most effective method of fatigue reduction, while pain is most effectively reduced with compression, massage, and cooling [3]. Mechanical compression, known as Intermittent pneumatic compression (IPC), has become increasingly popular in recent years, especially in athletes. A recent survey on professional soccer practitioners revealed that intermittent pneumatic compression was adopted by 57% of the teams surveyed [8]. The underlying assumption is that IPC increases blood circulation,, thus allowing for a faster regeneration due to improved tissue fluid exchange [9]. IPC devices involve various sleeves with inflatable air chambers, which are sequentially inflated to generate a peristaltic-like pressure pattern, causing fluids to flow towards the heart. Pneumatic compression devices are also applied in clinical practice (e.g., in venous thromboembolism prevention) [10]. Another example of circulation improvement therapy is intermittent negative pressure (INP). When using INP, the limbs are placed in a special chamber where oscillating negative pressure (vacuum) is generated to increase tissue perfusion. INP increases tissue perfusion and increases oxygen supply to the muscles [11].

Studies conducted so far are not unequivocal regarding the effectiveness of using variable pressure methods, and its application is varied. INP may present an innovative form of recovery, but research on the topic is limited [12–14]. Despite the wide use of compression, there is a lack of clear evidence for the benefit of tissue perfusion improvement or any comparison of various available methods. Meanwhile, such treatment is often commercially offered, and the offer may tempt athletes. Therefore, it is essential to provide more evidence on whether compression or vacuum treatment is effective.

The study aimed to verify the differences between INP and IPC repeatedly used over 48 h following strenuous eccentric exercise. We hypothesized that INP and IPC would not be superior to sham therapy in terms of alleviating pain and improving muscle function.

## Methods

### Participants

We conducted a randomised, controlled, double blind trial according to CONSORT reporting guideline [15] (Additional file 1).

67 healthy men were recruited between September 2018 and January 2019. The experimental data were gathered in the Laboratory of Biomechanics and Kinesiology and Laboratory of Diagnostics and Therapy of the Musculoskeletal System in the Regional Centre of Research and Development in Biala Podlaska. The total sample size was calculated with G ∗ Power (version 3.1.9.2; Germany), with the level of significance set at α = 0.05, power (1 − β) = 0.90 and effect size ƒ = 0.25 (ANOVA with repeated measures, within-between interaction). The total sample size determined by these calculations was 39 participants.

The inclusion criteria were: male; aged between 18 and 26 years; physically active (3–5 h weekly of recreational exercise such as gymnastics, functional training or team sports); no chronic disease or medication. The non-inclusion criteria were: sedentary lifestyle; lower limb or spine injuries in the year before the study; self-reported signs or symptoms of any current infection; any contraindications for INP or IPC. The exclusion criteria were: all injuries during the trial and failure to respect the protocol or evaluation schedules.

**Fig. 1:**
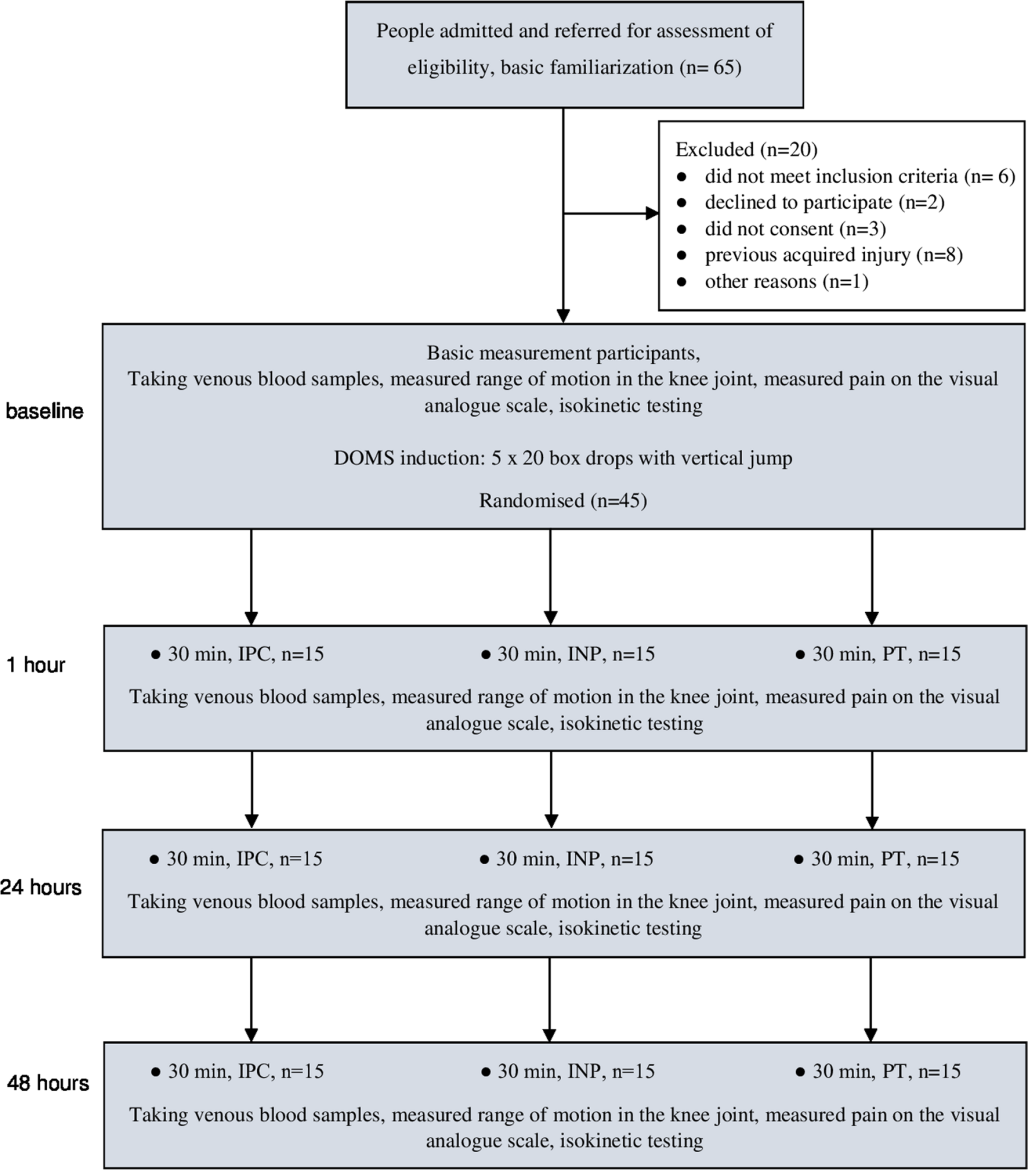
Design and flow of participants through the study. INP, intermittent negative pressure; IPC, intermittent pneumatic compression, PT, placebo therapy

Sixty-five men expressed their willingness to participate in the research. After assessment for eligibility and baseline measurements, twenty participants were excluded of the initial sixty-seven. Finally, forty-five men were allocated to the groups. Figure 1 shows the participants' flow through the study.

This research was conducted in accordance with the Declaration of Helsinki on human research. It was approved by the Research Ethics Committee, University of Physical Education, Warsaw, Poland (trial registration number SKE 01-02/2018). Participants were informed about the protocol (excluding procedures) before participating in this study. Written informed consent was obtained from all participants.

### Experimental procedures

An auditor who was not involved in the study performed block randomization using Research Randomizer (http://www.randomizer.org) and secured the group allocation details until the end of the trial. The auditor was not informed about the intervention used or the results' evaluation. Each measurement was carried out by a blinded assessor (qualified physiotherapist) who had no information about the treatment procedures. Participants were instructed not to disclose what type of intervention they had.

The participants were instructed not to take any medications and not perform any other therapeutic techniques or additional physical exercises during the data collection period. Physical activity during one week before the protocol study was also prohibited.

On the first day, venous blood samples were taken at rest to assess CK and LDH activity, baseline (PRE) measurements of muscle strength, range of motion (ROM), and visual analog scale (VAS). Measurements were made after at least 8 h of sleep and 2–3 h after the last carbohydrate meal. During the entire experiment, the participants could drink isotonic fluids ad libitum. After an initial 10-min warm-up with dynamic stretching elements (e.g. leg swings, arm circles, forward bends etc.), the participants performed the muscle-damaging protocol consisting of five sets of 20 box drop jumps from a height of 60 cm with 5-min rest breaks between sets. The participants were to step onto the box using alternating limbs, jump straight down, land on both legs, immediately perform a maximum vertical jump, and repeat the sequence every 5 s. Each participant was supervised by a volunteer tutor to control the participant's technique and repetition times. Ten minutes after the end of the drop jumps, the participants received a 30-min intervention according to group allocation. Five minutes after the intervention, a venous blood sample was collected and soreness, isokinetic muscle strength, and active knee joint flexion were measured. All therapies and measurements were repeated after 24 and 48 h.

The collected data was encoded and transferred to an external, blinded assessor to perform statistical analysis. Afterwards, the envelopes with allocation to groups were opened and assigned to the statistical analysis results.

#### Intervention

Immediately after eccentric exercises and after 24 and 48 h, participants performed either a session of (1) intermittent pneumatic compression (IPC) or (2) intermittent negative pressure (INP) or (3) sham microcurrent therapy (PT) for the lower body (legs and pelvis) according to group allocation. A duration of 30 min was set for all groups. INP was performed on Vacusport Regeneration System (Weyergans LTD, Germany), and IPC was performed on BOA-Max 2 (Metrum Cryo Flex, Poland). For INP, program number 2 was used, recommended by the manufacturer for recovery after exertion. Volunteers were placed inside the machine with the lower half of the body encased by a tubular pressure chamber. The device closing mechanism was tightened around the waist to assure that the chamber was sealed, allowing lower pipe pressure. The program consisted of five six-minute stages. In the first stage, vacuum to pause time was 7 to 7 s, with a vacuum of − 24.7 mmHg. In the second 8 to 6 s, (− 27.7 mmHg); in the third 8 to 8 s, (− 32.2 mmHg); in the fourth 9 to 9 s, (− 36.7 mmHg), and in the fifth 10 to10 s, (− 36.7 mmHg).

In the IPC group, the "sports massage" program was set. The participants were lying supine with the lower limbs and pelvis placed in 24-chamber compression sleeves. The ventricles' pressure was set for 80 mmHg. The selected program consisted of pumping three chambers in each leg simultaneously, starting from the lower limbs' distal parts. After chambers were inflated, the machine deflated all chambers simultaneously, and the next cycle began. Ten treatment cycles were set, with a 3-min duration each.

PT was subjected to sham microcurrent therapy (MET) using the Sonicator Plus 940 (Mettler Electronics Corporation. Anaheim, USA). Two current circuits were used (both lower limbs). Self-adhesive electrodes were placed on the quadriceps muscle. The procedure lasted 30 min without turning on the device. The participants had no insight into the control panel, and the device did not inform about the elapsed time with sound signals.

### Measures

#### Body measurements

Height was measured using a calibrated stadiometer (Seca 217, Seca GmbH&Co. KG, Germany). Body mass and fat percentage were determined with a body composition analyzer (Tanita, MC 718, Japan) using electrical bioimpedance.

#### Serum enzyme activity

Blood samples (6 ml) were collected from the antecubital vein in a sitting position into S-Monovette clotting tubes (Sarstedt, Germany). Tubes were left in an upright position for 30 min at room temperature, and the blood was then centrifuged (3000 g for 10 min at 4 °C) using a centrifuge (MPW med. instruments, Poland and the resulting serum was transferred to tubes, and stored at − 80 °C until analyzed. A certified laboratory diagnostician (KJ) analysed all samples to minimise as much as possible the influence of inter-assay variation.

Creatine kinase (CK, cat. No. C6412-060), and lactate dehydrogenase (LDH, cat. No. L6436-060) activity (U/L), were determined in serum using colorimetric spectrophotometry method at 340 nm wavelength at 37 °C using ready-made diagnostic reagents (Alpha Diagnostic, Poland) in an automatic biochemical analyzer A15 (BioSystems S.A., Spain). According to the manufacturer, CK intra-assay variation is 0.47% (sensitivity 1 U/L) and LDH intra- assay variation is 1.12% (sensitivity 4 U/L), with inter-assay variation of 0.62 and 1.65 respectively.

#### Pain assessment

The pain was assessed manually in the supine position by compressing a thumb pad (custom made 1.5 cm^2^ wooden circular probe) in the mid-distance between the anterior superior iliac spine and the patella's upper edge. The place of compression was marked on the first day with a semi-permanent marker. An assessor performed single compression with great accuracy, using the same compression force for 3 s, previously determined on a weight scale at the level of 10 kg ± 1 kg. A VAS was used to assess pain—the participants marked the level of pain sensation on a sliding colour scale (1–100 mm) immediately after releasing the pressure, and then the numerical values in millimetres were recorded [16].

#### Range of motion

The knee joint's ROM was measured using a handheld 360° range–14 inch metal goniometer (Fabrication Enterprises, USA). Measurements were made in the prone position. The stabilizing belt was put on at the height of the sacrum. The axis of rotation of the goniometer was set at the knee joint space level. One arm of the goniometer was aimed at the greater trochanter and the other arm at the fibula's lateral malleolus. The landmarks for subsequent measurements were then marked on the first day with a semi-permanent pen. Participants were asked to perform the maximum full flexion of the knee joint. Measurement of the knee joint angle was repeated three times, and the mean result was taken into account in further analysis.

#### Isokinetic muscle strength

Measurements were taken on an isokinetic dynamometer (Biodex Medical System 4-PRO, New York, USA). According to the manufacturer's protocol, the dynamometer was calibrated before each test session. Gravity correction was performed before each set of measurements. The participants performed a 5-min warm-up on a Monark LT2 cycle ergometer (Monark Exercise AB, Sweden) with a power of 120 Watts. Then in the next 5 min, they assumed a standard position on the chair and were stabilized with straps. All measurements were in concentric mode. The test consisted of a series of five extending and flexing movements (0°–90°). During the test, the participants were verbally encouraged to perform the movement to maximal effort. The isokinetic test was performed using fixed angular velocities of 60°∙s^−1^. H:Q ratio (concentric peak torque values of the hamstring to quadriceps muscle strength) was calculated to assess muscle strength balance [17]. In previous study the reliability of concentric peak torque using Biodex System was very high [18]. The intraclass correlation coefficients for quadriceps and hamstring were 0.93 and 0.89 respectively, with a standard error of measurement of 0.11 Nm and 0.09 Nm [18]. For further analysis, data from lower limbs were averaged.

### Statistical analysis

Data normality was tested by the Shapiro-Wilks test, and the sphericity assumption was confirmed by Mauchly's Sphericity test. Greenhouse–Geisser correction was employed to produce the F ratio, adjusting for lack of sphericity in a repeated-measures analysis of variance (ANOVA).

Changes over time were tested using repeated-measures two-way (time × group) ANOVAs followed by Tukey post hoc tests when appropriate. The significance level was set at *P* < 0.05. Data are expressed as means ± SD. Differences between means were expressed as mean with a 95% confidence interval (CI).

Effect sizes (η^2^) were calculated for the ANOVA main effects (time, group), and the magnitude of η^2^ was classified as small ≤ 0.06, moderate 0.07–0.14 or large > 0.15 [19]. All normally distributed analyses were performed using the Statistica ver. 13.3 (TIBCO Software Inc. Palo Alto, CA). LDH was not normally distributed, and Nonparametric Analysis for Longitudinal Data (nparLD Package 2.1, R-software, v.3.3.3) was used (ANOVA-type statistic, ATS) to compare main effects [20].

## Results

**Fig. 2:**
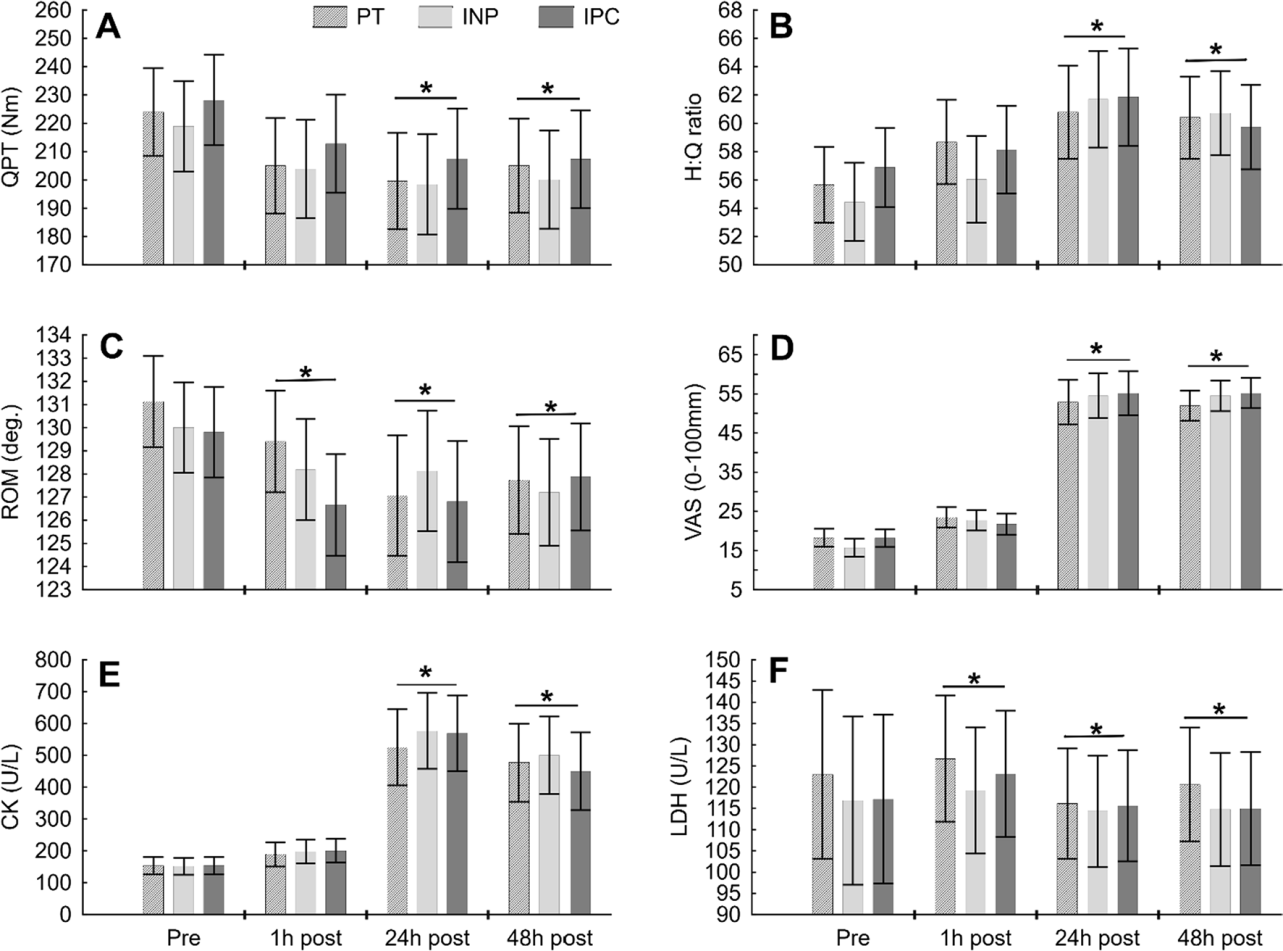
Mean ± SD values of: **A** Quadriceps peak torque; **B** Hamstring to quadriceps ratio; **C** Range of motion; **D** Visual analogue scale; **E** Creatine kinase; **F** Lactate dehydrogenase, on the studied groups. Indicates a significant difference between pre-exercise and post-exercise (*P < 0.05). INP, intermittent negative pressure; IPC, intermittent pneumatic compression; PT, placebo therapy

The groups were similar concerning anthropometric data (Table 1) and baseline measurements (Table 2). Results for all outcomes are presented in Fig. 2.

**Table 1:** Characteristics of participants (mean ± SD)

Variable	IPC	INP	PT
	(n = 15)	(n = 15)	(n = 15)
Age (year)	21.6 ± 3.2	21.8 ± 3.8	22.4 ± 2.9
Height (cm)	181.9 ± 4.8	181.5 ± 4.9	181.3 ± 5.9
Body mass (kg)	76.0 ± 7.4	75.0 ± 7.7	73.8 ± 7.3
BMI (kg/m^2^)	22.9 ± 1.5	22.6 ± 1.6	22.4 ± 1.6
FAT (%)	15.8 ± 2.9	16.0 ± 3.1	15.5 ± 2.6

BMI, body mass index; INP, intermittent negative pressure; IPC, intermittent pneumatic compression; PT, placebo therapy

**Table 2:** Mean ± SD values of the indices of muscle damage in the studied groups

Variable	PRE			1 h			24 h			48 h		
	IPC	INP	PT	IPC	INP	PT	IPC	INP	PT	IPC	INP	PT
CK (U/L)	154.3 ± 49.8	151.4 ± 55.8	152.9 ± 47.9	200.6 ± 67.6	198.4 ± 88.0	188.4 ± 60.9	568.5 ± 269.8	576.6 ± 209.7	525.2 ± 202.7	450.1 ± 228.1	500.6 ± 238.7	477.1 ± 237.6
LDH (U/L)	117.2 ± 31.2	116.8 ± 45.0	122.9 ± 36.8	123.2 ± 28.5	119.2 ± 24.9	126.6 ± 31.7	114.9 ± 25.3	114.7 ± 20.3	120.6 ± 30.3	115.7 ± 22.8	114.3 ± 18.2	116.1 ± 32.2
VAS (0–100 mm)	18.3 ± 4.8	15.7 ± 3.9	18.1 ± 4.0	23.5 ± 4.7	22.7 ± 5.6	21.7 ± 4.6	52.9 ± 11.3	54.5 ± 9.3	55.1 ± 10.7	52.0 ± 6.2	54.5 ± 7.7	55.2 ± 7.4
ROM (°)	129.8 ± 4.1	130 ± 4.0	131.1 ± 3.1	126.7 ± 4.3	128.2 ± 4.2	129.4 ± 4.2	126.8 ± 4.9	128.1 ± 4.7	127.1 ± 5.4	127.8 ± 4.5	127.2 ± 4.9	127.7 ± 3.9
QPT (Nm)	227.5 ± 33.0	222.8 ± 31.1	224.1 ± 26.5	212.0 ± 33.5	204.0 ± 31.9	205.0 ± 28.6	205.5 ± 31.4	198.1 ± 34.0	199.6 ± 31.0	207.4 ± 33.6	200.0 ± 30.9	205.0 ± 31.5
H:Q ratio	56.5 ± 4.4	54.7 ± 5.0	55.7 ± 5.8	58.1 ± 4.7	56.5 ± 5.0	58.7 ± 7.0	61.9 ± 4.4	61.7 ± 5.3	60.8 ± 8.1	59.5 ± 6.2	60.1 ± 5.8	60.4 ± 4.5

INP, intermittent negative pressure; IPC, intermittent pneumatic compression; PT, placebo therapy; CK, creatine kinase; LDH, lactate dehydrogenase; VAS, visual analogue scale; ROM, range of motion; QPT, quadriceps peak torque; H:Q ratio, hamstrings: quadriceps ratio

All procedures complied with the study protocol. Only eligible participants were randomized to the groups, and no assessors were unblinded during the study. The intention to treat approach was adopted in all analyses—all participants received the designated intervention and were analyzed in the group they had been randomly allocated. Due to the dynamometer's technical problem, isokinetic muscle strength testing for two participants (48 h post-exercise: INP n = 1; IPC n = 1) was infeasible. None of the subjects suffered during the research program, and no side effects were observed. Numerical data (Mean ± SD) are presented in Table 2. Mean (95% CI) difference within groups are presented in Table 3.

**Table 3:** Mean (95% CI) difference within groups for the indices of muscle damage in the studied groups

Variable	Difference within group Post 1 h minus pre-exercise			Difference within group Post 24 h minus pre-exercise			Difference within group Post 48 h minus pre-exercise		
	IPC	INP	PT	IPC	INP	PT	IPC	INP	PT
CK (U/L)	46.3 (6.7 to 85.8)	47.0 (7.4 to 86.5)	35.5 (− 4.1 to 75.1)	414.2 (307.8 to 520.6)	425.2 (318.8 to 531.6)	372.3 (265.9 to 478.7)	295.8 (172.0 to 419.6)	349.3 (225.4 to 473.1)	324.2 (200.4 to 448.0)
LDH (U/L)	6.0 (− 18.9 to 30.8)	2.4 (− 22.5 to 27.2)	3.8 (− 21.1 to 28.6)	− 2.2 (− 26.6 to 22.2)	− 2.1 (− 26.5 to 22.3)	− 2.3 (− 26.7 to 22.1)	− 1.5 (− 26.6 to 23.5)	− 2.5 (− 27.6 to 22.5)	− 6.8 (− 31.8 to 18.2)
VAS (0–100 mm)	3.6 (0.1 to 7.1)	7.1 (3.5 to 10.6)	5.2 (1.7 to 8.7)	37 (30.5 to 43.5)	38.8 (32.3 to 45.3)	34.6 (28.1 to 41.1)	37.1 (32.9 to 41.2)	38.8 (34.7 to 42.9)	33.7 (29.6 to 37.9)
ROM (°)	− 3.1 (− 5.8 to − 0.5)	− 1.8 (− 4.5 to 0.9)	− 1.7 (− 4.4 to 0.9)	− 3.0 (− 6.2 to 0.2)	− 1.9 (− 5.0 to 1.3)	− 4.1 (− 7.2 to − 0.9)	− 1.9 (− 4.5 to 0.6)	− 2.8 (− 5.4 to − 0.2)	− 3.4 (− 6.0 to − 0.8)
QPT (Nm)	− 15.5 (− 35.5 to 4.4)	− 18.8 (− 38.8 to 1.1)	− 19.1 (− 39.0 to 0.9)	− 22.1 (− 42.9 to − 1.2)	− 24.7 (− 45.6 to − 3.9)	− 24.5 (− 45.3 to − 36.6)	− 20.9 (− 41.7 to 1.3)	− 18.9 (− 44.3 to − 1.3)	− 19.1 (− 40.1 to 2.0)
H: Q ratio	1.6 (− 3.0 to 6.3)	1.8 (− 2.9 to 6.4)	3.0 (− 1.6 to 7.7)	5.4 (0.3 to 10.5)	7.0 (1.9 to 12.1)	5.1 (0.0 to 10.2)	2.9 (− 0.6 to 7.1)	6.3 (2.2 to 9.8)	4.7 (1.0 to 8.5)

INP, intermittent negative pressure; IPC, intermittent pneumatic compression; PT, placebo therapy; CK, creatine kinase; LDH, lactate dehydrogenase; VAS, visual analogue scale; ROM, range of motion; QPT, quadriceps peak torque; H:Q ratio, hamstrings: quadriceps ratio

### Creatine kinase and lactate dehydrogenase

The ANOVA showed neither significant main effect of group (F(2,42) = 0.17, *P* < 0.882, η^2^ < 0.1) nor interaction between time × group (F(6,126) = 0.239, *P* < 0.962, η^2^ < 0.1) for CK activity. There was an observed significant effect of time (F(3,126) = 87.16, *P* < 0.001, η^2^ = 0.68). Tukey’s post hoc testing shows a significant increase in CK activity from baseline to 24 h (*P* < 0.001) and 48 h (*P* < 0.001) and decrease for 24 – 48 h (*P* < 0.040). For LDH activity there was no significant effect neither for group (ATS = 0.15, *P* > 0.1) nor time and group (ATS = 0.41, *P* > 0.1). There was a significant effect of time (ATS = 2.07, *P* < 0.05). Post hoc testing shows significant differences (*P* < 0.05) in LDH activity PRE-1 h and 24–48 h after intervention.

### Visual analogue scale

There was a significant time effect increase in VAS score after exercise in all groups (F(3,126) = 429.9, *P* < 0.001, η^2^ = 0.91). VAS: PRE-1 h (*P* < 0.001), PRE-24 h (*P* < 0.001), PRE-48 h (*P* < 0.001) and VAS: 1–24 h (*P* < 0.001), 1–48 h (*P* < 0.001) with no difference between 24 and 48 h (*P* < 0.996). There was no group effect (F(2,42) = 0.177, *P* < 0.838, η^2^ < 0.1) and no interaction between time × group (F(6,126) = 0.94, *P* < 0.468, η^2^ < 0.1).

### Range of motion

There was a significant time effect in ROM (F(3,126) = 11.79, *P* < 0.001, η^2^ = 0.219) with no significant difference between groups (F(2,42) = 0.30, *P* < 0.743, η^2^ < 0.1) and no group × time effect (F(6,126) = 1.36, *P* < 0.236, η^2^ < 0.1). Significant decreases were observed in ROM PRE-1 h (*P* < 0.001), PRE-24 h (*P* < 0.001), PRE-48 h (*P* < 0.001), with no difference in ROM between 1 and 24 h (*P* < 0.528), 1–48 h (*P* < 0.817) and 24 -48 h (*P* < 0.964).

### Quadriceps peak torque

There was a significant time effect in peak torque (F(3,120) = 19.242, *P* < 0.001, η^2^ = 0.325) with no group effect (F(2,40) = 0.319, *P* < 0.728, η^2^ = 0.016) and no interaction between group × time effect (F(6,120) = 0.147, *P* < 0.989, η^2^ < 0.1). Significant decrease was observed in QPT: PRE-1 h (*P* < 0.001), PRE-24 h (*P* < 0.001), PRE-48 h (*P* < 0.001) with no difference in QPT 1–24 h (*P* < 0.335), 1–48 h (*P* < 0.785) and 24–48 h (*P* < 0.877).

### Hamstring to quadriceps ratio

There was a significant time effect in H:Q ratio (F(3,120) = 14.926, *P* < 0.001, η^2^ = 0.272) with no group effect (F(2,40) = 0.179, *P* < 0.836 η^2^ = 0.001) and no interaction between group × time effect (F(6,120) = 0.7535, *P* < 0.608, η^2^ = 0.036). Significant increases were observed in H:Q Ratio PRE-24 h (*P* < 0.001), PRE-48 h (*P* < 0.001) and 1–24 h (*P* < 0.001) and 1–48 h (*P* < 0.033) with no significant difference in H:Q Ratio PRE-1 h (*P* < 0.166), and 24 -48 h (*P* < 0.630).

## Discussion

To our best knowledge, this is the first study where INP and IPC performed daily for 48 h post eccentric exercise were compared in a randomized controlled trial. The research aimed to assess both therapies' potential impact on muscle recovery and soreness reduction in recreationally active men.

The results indicate no significant differences in recovery after exercise-induced muscle damage in INP or IPC versus placebo in any of the measured indicators of strength, pain, or joint mobility. The only factor that significantly influenced the recovery process was time. Most studies used a one-time compression session after muscle damage induced by exercise. For this reason, we decided to use the therapy several times over time to maximize its potential results, which is necessary for many therapeutic or sports situations where fast and effective recovery is essential.

### Serum markers of muscle damage

Increased serum CK activity is usually observed after intense eccentric exercise due to muscle microtrauma. CK activity reaches a peak within 24-48 h of muscle damage and subsequently declines if adequate rest is provided. However, many confounding factors influence both the base level of CK activity and changes in activity after muscle damage [21, 22]. We observed peak CK levels after 24 h and a slight decline afterwards, which is consistent with previous research [22]. No difference was observed between groups. Most previous research also identified no difference in CK levels using compression [23, 24] or negative pressure [12, 13]. A recent study demonstrated a more significant CK decrease after INP than after active recovery therapy. However, the participants were not blinded, and therefore a placebo effect cannot be ruled out [14]. The study was also carried out on professional soccer players after playing a full match, making it difficult to compare results. Peak LDH levels were observed 1 h after exercise, with a significant rise as soon as 1 h after exercise, similar to previous research. No difference was observed in LDH between the groups [25]. LDH was rarely measured in other studies on INP or IPC.

### Pain

Subjective perception of pain is a crucial complaint in DOMS, ranging from mild soreness to debilitating pain [1]. VAS is increased 1–4 days post-exercise. The peak usually occurs on the 2nd day, after which soreness declines. It is consistent with the present study, where VAS showed a sharp increase in the first 24 h and a very mild increase after 48 h in all groups. There was no difference in VAS increase with INP or IPC after 24 or 48 h. Most previous research yielded no statistically significant results, with no differences after applying IPC [24, 26, 27]. Fonda et al. (2015) described an improvement in VAS after INP, while van Rensburg et al. (2017) found no difference, similar to the present study [12, 13]. Cranston found IPC more effective than a sham procedure in flexor and extensor soreness reduction immediately after IPC and 24 h later in resistance-trained athletes [28]. The study design was similar to this study (similar observation time and intervention times) which suggests that perhaps IPC is more effective in resistance-trained athletes who generally recover faster, but not in recreationally active individuals.

### Range of motion

The range of motion has been demonstrated to be reduced in DOMS, possibly due to a shortening of the non-contractile muscle tissues and swelling due to an inflammatory response [1]. We observed significant ROM impairment after 1 h, 24 h, and 48 h from baseline, similar to another research [29, 30] There was no difference between the ROM in the INP, IPC, and PT groups. Few previous studies evaluated the effect of IPC or INP on ROM. Winke and Williamson (2018) described improvement in ROM of elbow flexion after IPC. However, the study was conducted on eight individuals and included constant passive compression over 5 days apart from IPC [31]. Therefore, no evidence currently indicates that INP, IPC or PT may improve ROM in DOMS.

### Strength

This study revealed a decrease in muscle voluntary contraction force (measured as QPT) after 1 h, 24 h, and 48 h, consistently with previous research on DOMS [27, 28, 32]. No effect of INP or IPC was found. Similar results indicating a lack of IPC effect were found in previous research [27, 28, 32]. A significant difference in strength was observed by Fonda et al. (2015), while van Rensburg et al. (2017) and Maior et al. (2020) did not compare strength in their studies [12–14]. Further research is required to determine whether and in what patient groups INP or IPC might be effective in muscle strength recovery in DOMS.

The underlying assumption is that mechanical pressure improves perfusion and accelerate recovery. The improved perfusion helps remove excess lactate but does not improve anaerobic performance [9]. Acute administration of IPC significantly upregulates PGC‐1α gene expression (by 77%) after 1 h following therapy, improving the muscle tissue's neovascularization [24]. Despite these theoretical benefits, it may be hypothesized that the intervention time is too short to yield any significant benefits, given that 30 min a day is only approximately 2% of the day. If the improved perfusion only occurs during the procedure, this period may be too short for any meaningful impact. Haun et al. (2017) applied IPC for 1 h periods and Northey et al. for 45 min, and neither observed any differences between IPC and control groups [24, 27]. Therefore, the application would need to be even longer if this hypothesis was true, which would be impractical, given that the limbs are immobilized for the duration of the treatment. It could be speculated that a combination of INP or IPC and passive compression in the remaining time might be effective, but new trials would be needed. Perhaps more frequent treatment instead of increasing the duration could improve muscle recovery's effectiveness after strenuous exercises.

## Conclusions

Intermittent pneumatic compression and intermittent negative pressure applied daily for 48 h were not superior to a placebo sham intervention in DOMS treatment induced by eccentric exercises. Further trials would be needed to determine whether more frequent or longer INP/IPC sessions might be more effective in muscle recovery.

## Supplementary Information

**Additional file 1**. CONSORT 2010 checklist.

## Data Availability

The datasets generated and/or analysed during the current study are not publicly available due to limitations of ethical approval involving the patient data and anonymity but are available from the corresponding author on reasonable request.

## Abbreviations

IPC: Intermittent pneumatic compression
INP: Intermittent negative pressure
PT: Sham microcurrent
CK: Creatine kinase
LDH: Lactate dehydrogenase
ROM: Range of motion
VAS: Visual analogue scale
QPT: Quadriceps peak torque
H:Q ration: Hamstring to quadriceps ratio
